# Overexpressing natural killer group 2 member A drives natural killer cell exhaustion in relapsed acute myeloid leukemia

**DOI:** 10.1038/s41392-025-02228-5

**Published:** 2025-05-05

**Authors:** Juan Xie, Xue-Fei Liu, Tong Zhou, Long Liu, Rui-Qin Hou, Xing-Xing Yu, Ze-Ying Fan, Qian-Nan Shang, Ying-Jun Chang, Xiao-Su Zhao, Yu Wang, Lan-Ping Xu, Xiao-Hui Zhang, Xiao-Jun Huang, Xiang-Yu Zhao

**Affiliations:** 1 Peking University People’s Hospital, Institute of Hematology, Beijing Key Laboratory of Cell and Gene Therapy for Hematologic Malignancies, National Clinical Research Centre for Hematologic Disease; No. 11 South Street of Xizhimen, Xicheng District, 100044 Beijing, China; 2 Peking-Tsinghua Center for Life Sciences, Peking University, 100871 Beijing, China; 3 Peking University People’s Hospital department of blood transfusion, No. 11 South Street of Xizhimen, Xicheng District, 100044 Beijing, China

**Keywords:** Tumour immunology, Haematological cancer

## Abstract

Acute myeloid leukemia (AML) relapse is associated with poor prognosis. While natural killer (NK) cell therapy can induce leukemia remission, infused NK cells are prone to exhaustion. Elucidating the molecular mechanisms driving NK cell exhaustion in AML patients could provide critical insights for developing novel strategies to optimize NK cell-based immunotherapies. In this study, we systematically investigated NK cell exhaustion in relapsed AML patients following allogeneic hematopoietic stem cell transplantation (allo-HSCT) through phenotypic assessments, functional assays, and RNA sequencing analyses. Compared to NK cells from complete remission patients and healthy controls, NK cells from relapsed AML patients exhibited an exhausted phenotype, marked by reduced maturity, elevated expression of the inhibitory receptor NKG2A, impaired cytotoxicity, and suppression of the PI3K-AKT pathway. Notably, NKG2A expression levels on NK cells correlated with disease progression. Blockade or genetic knockout of NKG2A effectively reversed NK cell exhaustion both in vitro and in an AML mouse model. Furthermore, activation of the PI3K-AKT pathway significantly enhanced cytotoxicity in exhausted NK cells. We found that excessive activation of the NKG2A/HLA-E axis was associated with PI3K-AKT pathway inhibition, and blocking the NKG2A/HLA-E interaction or knocking out NKG2A restored AKT phosphorylation in exhausted NK cells. In summary, AML cells drive NK cell exhaustion through overactivation of the NKG2A/HLA-E axis and suppression of the PI3K-AKT pathway. Targeting the NKG2A/HLA-E axis represents a promising therapeutic approach to restore PI3K-AKT signaling and reverse NK cell exhaustion.

## Introduction

Acute myeloid leukemia (AML) is the most common form of acute leukemia in adults,^1^ 35–45% of patients under 60 years attain long-term survival, this proportion precipitously drops to 10–15% in those beyond 60 years of ages. Immunotherapeutic approaches, particularly immune checkpoint inhibitors, chimeric antigen receptor (CAR) T-cell therapy, and adoptive natural killer (NK) cell infusion, have demonstrated significant clinical efficacy across multiple cancer.^2,3^ However, immunotherapy was limited applyied in AML due to the disease heterogeneity, lack of AML-exclusive antigen and the complex immunosuppressive microenvironment.^4,5^ Allogeneic hematopoietic stem cell transplantation (allo-HSCT) remains an effective therapeutic intervention for acute myeloid leukemia (AML); however, the relapse persists as a major risk factor contributing to transplant failure and adversely impacting patients survival outcomes.^6^ Consequently, understanding the characteristics of immune cells during AML progression will benefit for increasing the efficiency for immunotherapies.

NK cells are the first reconstituted lymphocytes and play a role in immunosurveillance of leukemia after allo-HSCT.^7,8^ NK cell dysfunction has been extensively studied in primary AML patients, characterized by reduced cytotoxicity and downregulation of activating receptors such as NKp30, NKp46, and NKG2D.^9–12^ Notably, leukemic cells lacking NKG2D ligands can evade NK cell-mediated killing,^13^ and restoring NKG2D ligands on leukemic cells through PARP1 inhibition can enhance NK cell anti-tumor activity in vivo. Additionally, TGF-β1 secreted by CD4⁺ T cells induces dysfunction in bone marrow-derived NK cells in early-stage relapsed AML patients post-HSCT.^14^ The transcriptomic landscape of bone marrow NK cells underwent substantial alterations mediated by AML cells in which the effector molecules of NK cell were significantly downregulated.^15–17^ Recently studies have demonstrated that the TGF-β released by AML blasts helped myeloid blast cells to evade the NK cells immune surveillance, in which resulted the NK cells dysfunction.^18^ Moreover, the impairment of NK cells was associated with the epigenetic reprogramming driven by the core transcript factor, BATF, knockout the BATF protected NK cells from TGF-β drived immunosuppressive action and in turn increased NK cell cytotoxicity function. However, NK cell dysfunction encompasses various states, including senescence, suppression, and exhaustion.^19–21^ Similar to T cell exhaustion, NK cells can become exhausted under chronic antigen stimulation,^15,16,19,22,23^ and the blockade of immnune checkpoint is efficient to overcome the dysfunction of NK cells. Therefore, we hypothesized that consistent leukemia stimulation could induce NK cell exhaustion and NK cell exhaustion could be reinvigorated once remission was achieved in leukemia patients. There are several studies reported the inhibitory receptors, such as TIGIT,^24,25^ Tim3,^26^ PD-1,^27^ and NKG2A,^28^ were involved in NK cell dysfunction in AML. NKG2A is an important receptor for NK cell education and improves responsiveness of NK cell^29^ while the NKG2A expression is variable during NK cell development. HLA-E is the ligand of NKG2A, which is overexpressed on tumor cells, macrophages and dendritic cells (DCs),^30^ the inhibitory signaling mediated by the interaction of NKG2A and HLA-E has been identified in many tumors.^30,31^ Blocking the NKG2A/HLA-E pathway between NK cells and tumors has been proposed as a potential strategy to improve NK cells anti-tumor efficiency in many tumors, including AML,^32^ glioblastoma,^33^ pancreatic ductal adenocarcinoma (PDAC)^34^ and lymphoma.^35,36^ However, it remains unclear whether NK cells exhaustion can be induced by overactivated NKG2A/HLA-E interaction. The phosphatidylinositol-3-kinase (PI3K)-AKT pathway orchestrates essential biological programs in NK cells, including their development, maturation, priming, and cytotoxic functions.^37,38^ Abnormal PI3K-AKT signaling decreased NK cell cytotoxicity function against tumors.^26,39,40^ The inhibitory receptor NKG2A interacts with HLA-E to suppress NK cell activity through SHP-1 and SHP-2 signaling. Genetic ablation of NKG2A results in diminished phosphorylation of these downstream phosphatases. Consequently, NKG2A-deficient NK cells exhibit heightened activation status, characterized by enhanced phosphorylation of VAV1 and ERK1/2, which are critical mediators of cytotoxic degranulation. SHP-1 inhibition could affect the AKT-β-catenin pathway.^41^ The potential crosstalk between the NKG2A/HLA-E axis and PI3K-AKT signaling pathway in NK cells remains an unresolved aspect of NK cell biology.

Investigating the effect of the NKG2A/HLA-E interaction and the PI3K-AKT pathway for the NK cell exhaustion may provide critical insights into AML immune evasion and reveal novel therapeutic strategies to impede disease progression. In this study, we identified distinct phenotypic and functional alterations in NK cells from relapsed AML patients, whose NK cells impaired in cellular maturation, upregulated expression of inhibitory receptors, and significantly reduced the cytotoxic activity. Moreover,the transctiption factors including T-bet and eomes were also decreased. We discovered that the HLA-E is highly expressed in relapsed patients,which is associated with the exhaustion of NK cells via excessive NKG2A/HLA-E interaction. The PI3K-AKT pathway was significantly inhibited in exhausted NK cells. Blockade or knockout of the NKG2A could effectively increase activation of PI3K-ATK pathway and enhance NK cell anti-tumor function both in vivo and in vitro. Collectively, our study elucidates that excessive interaction of the NKG2A-HLA-E axis mechanistically contributes to NK cell exhaustion through suppression of the downstream PI3K-AKT signaling pathway, hinted that targeting NKG2A/HLA-E axis is a promising therapeutic method enhancing NK cell functionality and reversing the exhausted phenotype in the future.

## Results

### NK cell is exhausted in relapsed AML patients post-HSCT

After chronic stimulation, exhausted NK cells are characterized by poor effector functions, increased expression of inhibitory receptors and decreased expression of activation receptors, as indicated by the downregulated expression of the transcription factors T-bet and eomesodermin (Eomes). However, whether NK cells are exhausted in AML patients who relapse after allo-HSCT has rarely been determined. In our study, we enrolled 60 patients (30 who relapsed and 30 who achieved CR) and 12 healthy donors as controls to study whether there existed NK cells exhaustion in relapsed AML post allo-HSCT (Supplementary Table 1).

**Fig. 1:**
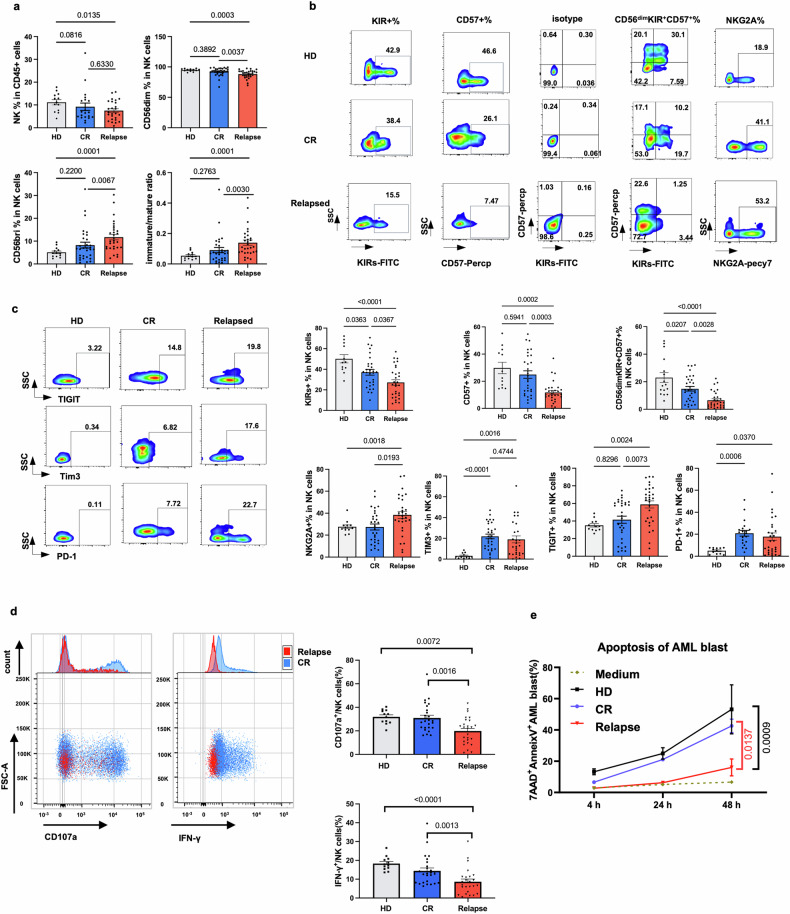
BMNK cell phenotype of AML patients after HSCT. **a** Frequency of BMNK cells in leukocyte, frequencies of CD56^bright^ and CD56^dim^ cells and the ratio of immature to mature NK cells. **b** Representative plots (top) of CD57^+^, KIRs^+^, CD56^dim^KIRs^+^CD57^+^, NKG2A^+^ BMNK cells from healthy controls (HD, *N* = 12), complete remission patients (CR, *N* = 30) and relapse patients after HSCT (Relapse, *N* = 30), and frequencies (bottom) of CD57^+^, KIRs^+^, CD56^dim^KIRs^+^CD57^+^. **c** Representative plots (left) and the expression level (right) of NKG2A, TIM-3,TIGIT and PD-1on NK cells from healthy controls (HD, *N* = 12), complete remission patients (CR, *N* = 30) and relapsed patients (Relapse, *N* = 30) after HSCT (right). **d** Representative plots (left) and summary data showing the IFN-γ production and degranulation capacity of CD107a of BM NK cells after treatment with K562 cells for 4 h. CR, *N* = 26; relapse, *N* = 26; HD, *N* = 12 (right). **e** The 7AAD^+^Annexin V^+^ cells (apoptotic leukemia cells) induced by BMNK cells sorted from healthy donors(HD) (*N* = 3), CR (*N* = 3) or relapse (*N* = 3)

The frequency of NK cells in leukocyte in relapsed patients was lower than that in healthy donors but comparable to that in CR patients (Fig. 1a). Furthermore, the frequency of CD56^dim^ mature NK subsets was lower in patients relapsed with AML than healthy donors and CR patients while the frequency of CD56^bright^ immature NK cells was increased in relapsed patients. In addition, we compared the ratio of immature/mature NK cells among three groups and found that it was significantly increased in relapsed patients but was comparable between CR and healthy donors (Fig. 1a). In addition, the expression of terminal differentiation markers, such as CD57 and KIRs (including KIR2DL1, KIR2DL2/L3, and KIR3DL1), were downregulated in relapsed patients (Fig. 1b). Compared to healthy donors, the population of the most mature NK cells (CD56^dim^KIR^+^CD57^+^) was reduced in patients with CR and further declined in those who had relapsed, which indicated that the maturation of NK cells may be impaired in patients undergoing HSCT than healthy donors, and NK cells maturation may be even worsen with disease progression.

We conducted further analysis on the profiles of activating and inhibitory receptors expressed on NK cells. Gating strategy was exhibited in Supplementary Fig. 1a. Interestingly, we found that the frequencies of the activating receptors NKp30,NKp46 and NKG2D were comparable among the three groups (Supplementary Fig. 1b). However, it is worth noting that the mean fluorescence intensities (MFIs) of NKp30 was increased in relapsed patients compared to healthy donors. Additionally, the MFI of NKp46 in relapsed patients was even higher than that in CR patients (Supplementary Fig. 1b). Furthermore, we observed a significant alteration in the inhibitory receptor profile of relapsed AML patients (Fig. 1c). Specifically, NKG2A was found to be highly expressed in relapsed patients compared to those in complete remission (CR), mirroring the trend seen in TIGIT expression. Moreover, the expression of Tim3 and PD-1 on NK cells was comparable between CR patients and relapsed patients but significantly higher than that of healthy donors (Fig. 1c). Given that all patients in our cohort were CMV positive, we also assessed the levels of NKG2C receptor among the three groups. Interestingly, in comparison to those from healthy donors, the frequency and MFI of NKG2C were found to be higher in CR patients as well as in relapsed patients (Supplementary Fig. 1b).

The observed decreased maturation and increased expression of inhibitory receptors in relapsed patients led us to investigate whether NK cells experience cytotoxicity dysfunction during disease progression. Consequently, we assessed the cytotoxicity as well as cytokine release of NK cells against K562 cells or AML blast. Our results revealed that the levels of IFN-γ and CD107a expression in NK cells responding to K562 cells were significantly lower in relapsed patients compared to CR patients (Fig. 1d). The rate of apoptosis in AML blasts induced by NK cell-mediated cytotoxicity was found to be decreased in relapsed patients compared with CR patients or healthy donors (Fig. 1e). Collectively, we determined that NK cells were exhausted in relapsed AML patients post-HSCT with the characteristic of less maturity, highly expressed inhibitor receptors and cytotoxicity dysfunction.

### Long-term AML cell stimulation induces the NKG2A overexpression and NK cell exhaustion

**Fig. 2:**
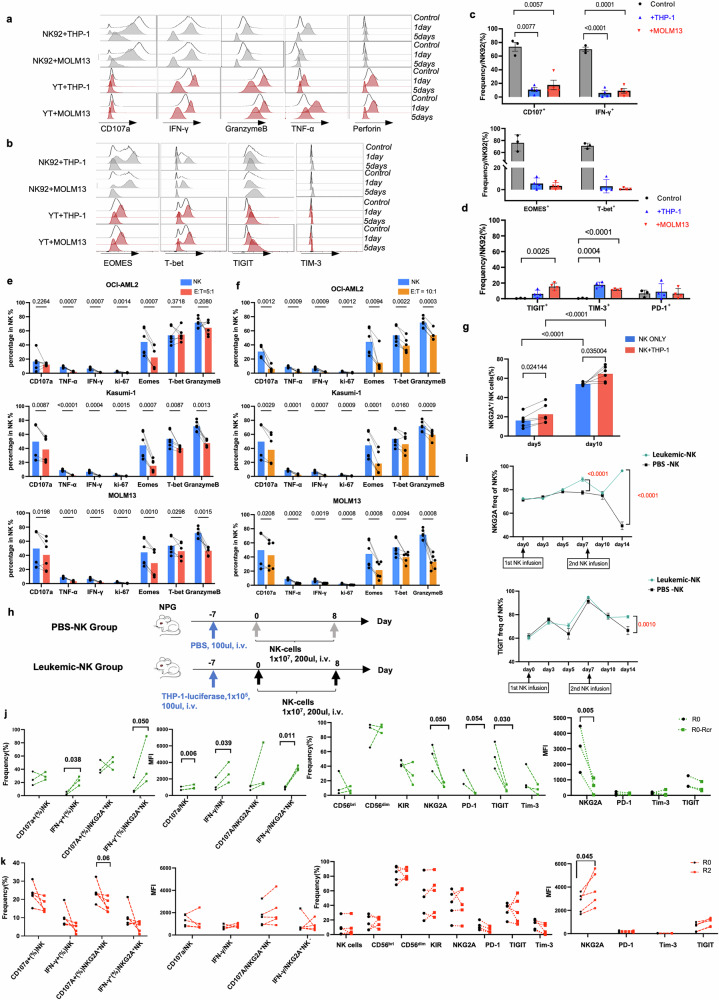
AML induces NK cell exhaustion. **a**, **b** Representative MFI results for NK92 or YT cell cytotoxicity, phenotype and transcription factor expression after coculturing with medium (control), MOLM13 or THP-1 cells for 1 day or 5 days at 10:1 E:T ratio. **c** The frequencies of cytokine release (CD107a and IFN-γ) (top) and the expression of the transcription factors T-bet and Eomes in NK92 cells after coculture (bottom) with leukemia cell lines. **d** The expression of inhibitor receptors in NK92 cells after coculture with leukemia cell lines. **e**, **f** cytotoxicity function of NK cells expression after coculture with OCI-AML2 and Kasumi-1 cells for 5 days under the E:T ratio of 5:1(**e**, left) and 10:1(**f**, right). **g** The expression of primary NK cells co-cultured with THP-1 cells for 5 and 10 days at the E:T ratio of 5:1 (**h**) Illustration of THP-1- luciferase AML mice model injected with PBS group and NK group (*n* = 12). **i** The dynamic expression of NKG2A and TIGIT in mice model injected with NK cells in PBS-NK and Leukemic NK groups (*n* = 12). **j** The function (left) and phenotype (right) of BMNK cell from patients from relapse to CR in AML patients post HSCT. R0 represents the first relapse stage, and Rcr represents the remission stage (*N* = 3). **k** The frequency of NK cells in leukocytes and the dynamic the phenotype characteristics in patients between relapsed and refractory relapse status. R2 represents the recurrent relapse stage(*N* = 5)

Considering chronic stimulation could induce NK cells exhaustion, we hypothesis that long-term leukemia blast stimulation could trigger NK cells exhaustion in vitro. To addressed this issue, we initially cocultured NK cell lines (YT or NK92 cells) with AML cell lines in vitro. It was observed that shor-term coculture (24 h) did not significant impact on the functional capacity of NK cells (Fig. 2a); however, after 5 days coculture with THP-1 cells, the CD107a and IFN-γ were decreased significantly (Fig. 2a, b). Moreover, T-bet and Eomes expression in NK92 cells were markedly reduced (Fig. 2c, Supplementary Fig. 2a). However, the level of inhibitory receptors, including TIM-3, and TIGIT were increased significantly (Fig. 2d).

Meanwhile, primary NK cells were cocultured with AML cell lines (Kasumi-1, MOLM13 and OCI-AML2) at different E:T ratios (5:1 and 10:1) to determine that long term AML cells stimulation would induce the NK cells exhaustion directly. Results showed after co-culturing with AML cell lines for 5 days, the cytotoxicity function and the expression of transcription factors in primary NK cells were significantly decreased (Fig. 2e, f). Furthermore, we noticed that the expression of NKG2A was persistently increased with the time of coculture gradually which higher after 10 days of coculture (Fig. 2g, Supplementary Fig. 2b). Moreover, in the AML cell line xenograft mice model, we demonstrated that sustained exposure to leukemic cells in vivo induces functional exhaustion of NK cells, as evidenced by comprehensive phenotypic and functional analyses (Fig. 2h). The expression of TIGIT and NKG2A of NK cells post NK cells infusion were significantly increased with tumor progression in THP-1 tumor bearing NPG mice (Fig. 2i).

As NK cell exhaustion was significantly elevated in relapsed AML patients, we hypothesis this exhaustion resulted from prolonged AML cell stimulation in vivo. We conducted follow-up assessments of NK cell exhaustion and functional capacity in two cohorts: relapsed patients (R1) who were responsive and achieved remission after treatment (Rcr) and those with persistent relapse post- treatment(R2). In responsive patients (Rcr), we observed a significant reduction in the levels of inhibitory receptors on NK cells, accompanied by increased cytokine production simultaneously (Fig. 2j). However, in recurrent relapse patients (R2, *n* = 5), the expression of NKG2A was increased, and the cytotoxicity of NK cells was slightly decreased (Fig. 2k). Our findings suggest that prolonged AML cell stimulation leads to NKG2A overexpression, which is associated with NK cell exhaustion.

### Overexpression of HLA-E on AML blast is correlate with NK cell exhaustion in relapsed AML patients

To investigate the potential mechanism of NK cell exhaustion in patients experiencing relapse due to AML cell infiltration, we conducted further analysis on the ligand profiles of inhibitory and activating receptors on CD34^+^CD45^dim+^ BMNCs from healthy donors, CR patients, and relapsed patients (Supplementary Fig. 3). Our findings revealed a decrease in HLA-ABC expression in relapsed patients compared to healthy donors (Supplementary Fig. 4a). Additionally, there was a significant increase in HLA-E expression both in relapsed and CR patients compared to healthy donors, which correlated with the elevated NKG2A expression among these three groups (Supplementary Fig. 4b). Furthermore, PD-L1 expression was higher in CR and relapsed patients (Supplementary Fig. 4c). Moreover, the expression of CD155 and CD112, ligands of TIGIT, were comparable between CR and relapsed patients (Supplementary Fig. 4d). Notably, CR patients exhibited higher ULBP2/5/6 expression on AML blast cells than both relapsed patients and healthy donors while MICA/B ligands were comparable, suggesting a more activated NKG2D-ligands axis in CR group than relapsed patients.

Considering there existed AML relapse post allo-HSCT, we hypothesized that there might be some unique immune escape mechanisms in relapsed AML blast compared to new diagnosis AML patients. However, the differences of ligand profile between primary and relapsed AML patients remains poorly understood. As the significant differences in tumor burden and phenotype of AML cells between these two groups, we conducted an assessment of ligand expression on CD34^+^CD117^+^CD33^+^CD13^+^ AML blast in both primary AML patients and relapsed patients. Our findings revealed a sharp decrease in HLA-ABC expression in primary AML patients compared to relapsed patients (Supplementary Fig. 4e), while higher levels of HLA-E and PD-L1 expression were detected in relapsed AML patients than in primary AML patients (Supplementary Fig. 4f-g). These results suggest that ligand-receptor interactions may play a role in AML relapse, particularly the interaction between NKG2A and HLA-E.

It is reported that the IFN-γ produced by T and NK cells in newly diagnosed AML patients can upregulate the cell surface expression of HLA-E^42^ and is related to venetoclax resistance.^43^

Thus, we aimed to investigate whether inflammatory cytokines were upregulated in relapsed patients, leading to the overexpression of HLA-E on AML cells. Cytokine levels in the BM supernatant from compared healthy controls, CR and relapsed AML patients were quantified. The analysis revealed a significant increase in IL-10, TNF-α, and IFN-γ levels in plasma among relapsed patients (Supplementary Fig. 4h). Furthermore, it was determined that the individual cytokines of TNF-α, IL-10 or IFN-γ or the combinations of cytokines could significantly upregulate HLA-E expression in AML blast cells (Supplementary Fig. 4i). Meanwhile, the cytokines that activated NK cells including IL-21, IL-18, and IL-15 were also found to be much higher in relapsed patients compared to CR and healthy donors (Supplementary Fig. 4j).

Overall, our data suggest that stimulation with multiple inflammatory cytokines induces high expression of HLA-E on leukemia blast which might contribute NK cells exhaustion through NKG2A/HLA-E interaction in relapsed AML patients post allo-HSCT.

### NKG2A/HLA-E axis blockade boosts the anti-AML activity of NK cells

**Fig. 3:**
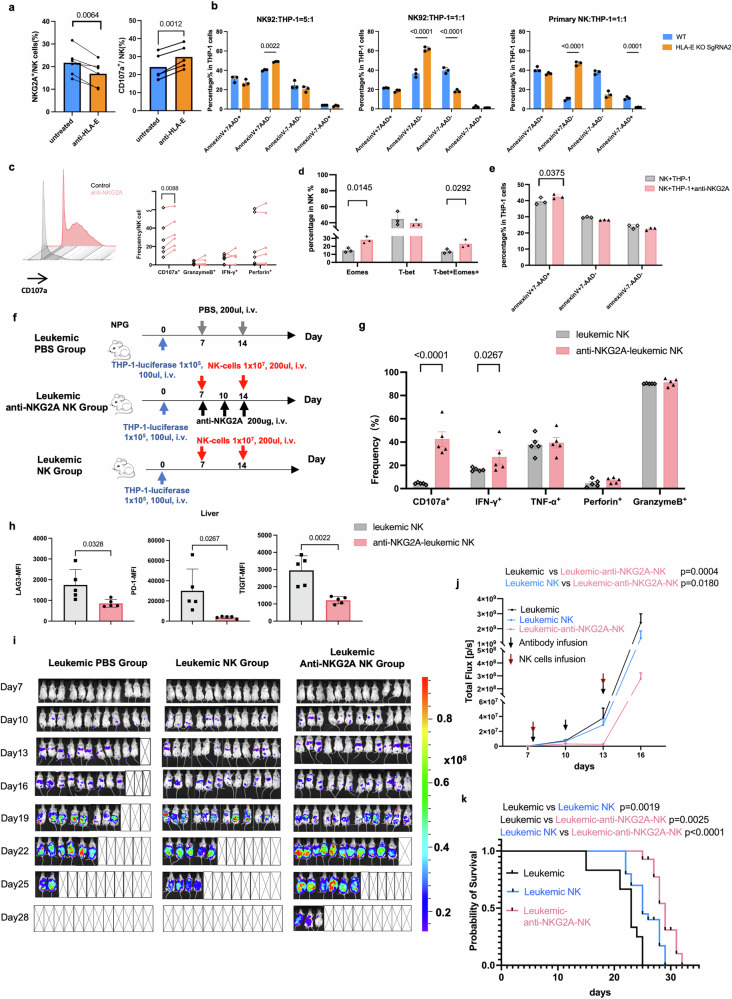
NK cell exhaustion was rescued by blocking the NKG2A/HLA-E axis. **a** THP-1 cells were pretreated with anti-HLA-E and then cocultured with primary NK cells for 5 days, the anti-HLA-E treatment decreased NKG2A expression on NK cells (left, *N* = 6) and increased CD107a+ NK cell subsets (right, *N* = 5). **b** HLA-E knockout THP-1 cells were cocultured with NK92 or primary NK cells for 6 h, and then the Annexin V and 7-AAD were utilized for apoptosis analysis (*N* = 3) under 1:1 and 5:1 E:T ratio. **c** Anti-NKG2A increased NK cell CD107a expression in vitro (*N* = 6). **d** Anti-NKG2A increased NK cell Eomes and T-bet+Eomes+ NK subsets in vitro (*N* = 3). **e** Anti-NKG2A treated NK cells increase the killing effect against THP-1 cells in vitro (*N* = 3). **f** AML mice model injected with leukemic THP-1 cells (Leukemic PBS group, *N* = 12) or with NK cells (Leukemic NK group, *N* = 12), or with anti-NKG2A treatment on NK cells (leukemic anti-NKG2A NK group, *N* = 12). **g** The cytotoxicity of NK cells between leukemic NK group and leukemic anti-NKG2A NK group in vivo. **h** The inhibitory receptors expressed on liver-derived NK cells including LAG3,PD-1 and TIGIT among PBS NK, Leuk-NK and Leukemic anti-NKG2A NK groups. living imaging (**i**) and total tumor flux (**j**) comparison among the PBS group (*N* = 12), leukemic NK cell group (*N* = 12) and leukemic anti-NKG2A NK group(*N* = 12). **k** Kaplan‒Meier curves and log-rank test results for overall survival among three groups (*N* = 12)

Given that excessive interaction of NKG2A and HLA-E was associated with NK exhaustion, and blockade of NKG2A has been demonstrated to enhance NK cell-mediated cytotoxicity against solid tumors,^35^ we further investigated whether incorporating the NKG2A-blocking antibody monalizumab or HLA-E blocking antibody could mitigate NK cell exhaustion in AML. Firstly, we blockaded the HLA-E on THP-1 cells to determine the efficacy in increasing NK cell function. Results showed that blocking HLA-E could effectively decrease the expression of NKG2A and enhance the expression of CD107a in primary NK cells against THP-1 cells (Fig. 3a). Furthermore, the HLA-E of target cells was knocked out and then co-cultured them with NK cells to detect whether the cytotoxicity function of NK cells on target cells could be elevated. Results indicated that the ability of NK92 cells and primary NK cells to kill HLA-E KO THP-1 target cells was significantly enhanced compared with that of the wild type THP-1 cells, suggesting that the blockade of the NKG2A/HLA-E pathway increased the function in NK cells (Fig. 3b).

Moreover, the NKG2A antibody was utilized to determine the efficacy of blocking NKG2A in increasing NK cell function. Results showed the addition of anti-NKG2A antibody significantly enhanced the expression of CD107a on NK cells against THP-1 cells (Fig. 3c), accompanied by elevated expression of transcription factors (Fig. 3d). Furthermore, NK cells subjected to NKG2A blockade exhibited markedly improved cytotoxic activity against target cells induced higher apoptosis of target cells (Fig. 3e).

In addition, we constructed the leukemia NPG mouse model by THP-1-luciferase cells, and 7 days later expanded NK cells were infused with or without an anti-NKG2A antibody (Fig. 3f). Results showed that the splenic NK cell cytotoxicity against THP-1 cells was significantly enhanced following anti-NKG2A blockade in vivo (Fig. 3g). We also tested the inhibitory receptors expression of NK cell from mice tissues at day 15. Blocking NKG2A decreased the LAG3, PD-1 and TIGIT expression at day 15 in liver NK cells (Fig. 3h). Remarkably, compared to PBS or NK cells infusion alone in leukemic mice models, adoptive NK cells infusion combination with 3 doses of anti-NKG2A led to a prolonged delay in tumor growth (Fig. 3i, j) and significantly increased survival(Fig. 3k). Our results identified that blocking the NKG2A reinvigorated NK cells from exhaustion in vivo.

### NKG2A knockout reinvigorats NK cells from exhaustion

**Fig. 4:**
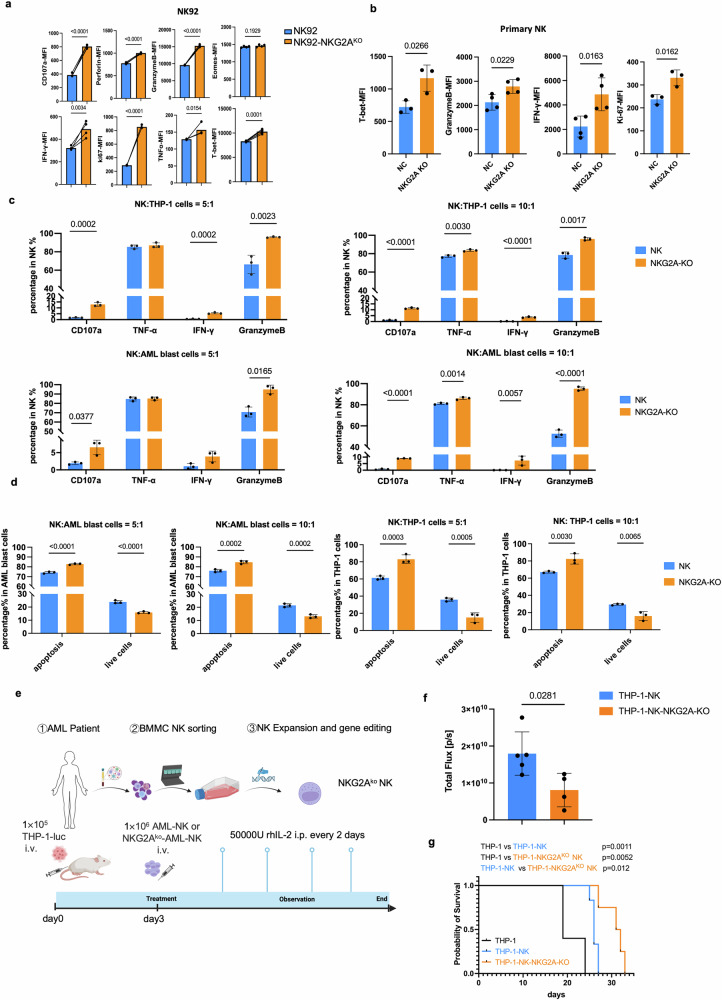
NKG2A knockout reinvigorated NK cells from exhaustion. **a** CD107a, perforin, GZMB, IFN-γ, Ki67, TNF-α, T-bet, and Eomes expression in NKG2A knockout and wild-type NK92 cells after stimulation with THP-1 cells. **b** The T-bet, GZMB, IFN-γ and Ki-67 expression on NKG2A knockout primary NK cells stimulation with THP-1 for 5days (*N* = 3). **c** The AML derived NKG2A-knockout NK cells were stimulated with THP-1 and AML blast cells for 6 h to detect the cytotoxicity function under 5:1 (left) or 10:1 (right) E:T ratio. **d** The apoptosis rate of THP-1 cells or AML blast cells induced by AML-NK cells and NKG2A KO NK cells. **e** The schematic diagram generated from online tools bioRender, the AML mice models infusion with AML derived NKG2A KO cells or AML derived wild type NK cells (*N* = 5). **f** The total tumor flux of AML mice model at day 23 post THP-1 cells infusion between NK and NKG2A KO groups (*N* = 5). **g** Survival curve of THP-1 with PBS, THP-1 with NK cells infusion and THP-1 with NKG2A-KO NK cells groups (*N* = 5)

In order to find out whether NKG2A would be efficient to improve NK cell function and reinvigorate NK cells from exhaustion, we firstly used the Cas9-RNP proteins to knockout the KLRC1gene in NK92 cell and primary NK cell. The knockout of NKG2A (NKG2A-KO) in NK92 cell effectively enhanced the cytotoxicity compared to the negative control group (Fig. 4a).

Additionally, it was also determined that whether the NK cell exhaustion could be alleviated in NKG2A KO primary NK cells when co-cultured them with THP-1 cells. NKG2A KO NK cells elevated expression of T-bet, as well as increase in IFN-γ release, expression of GZMB and Ki-67 after 5 days of stimulation with THP-1 cells (Fig. 4b). Lower LAG3 expression was found in NKG2A KO primary NK cell (Supplementary Fig. 5a).

It has not been determined that whether NKG2A knockout will be efficient to improve the AML patients derived NK cells function. NKG2A-deleted NK cells exhibited markedly increased higher expression levels of TNF-α, perforin, IFN-γ, granzyme B, and CD107a compared to wild-type NK cells (Fig. 4c). The killing assay results suggested that NKG2A KO primary NK cells were much powerful to kill cells highly expressed HLA-E (the THP-1 cells and AML blast cells) which induced higher rate of apoptosis of target cells after 6 hours co-culture(Fig. 4d, Supplementary Fig. 5b), suggested that NKG2A knockout effectively increased the AML derived NK cells cytotoxicity function.

Furthermore, we infused the AML derived-NK cells with or without NKG2A KO into the THP-1 CDX mice model (Fig. 4e). The results showed that NKG2A KO group achieved lower tumor burden (Fig. 4f, Supplementary Fig. 5c) and better survival (Fig. 4g) than other groups. In summary, all of these results suggest that NKG2A knockout is an optimal method to restore NK cells function in AML patients.

### PI3K-AKT signaling pathway inhibition involves the NK cell exhaustion in AML patients

**Fig. 5:**
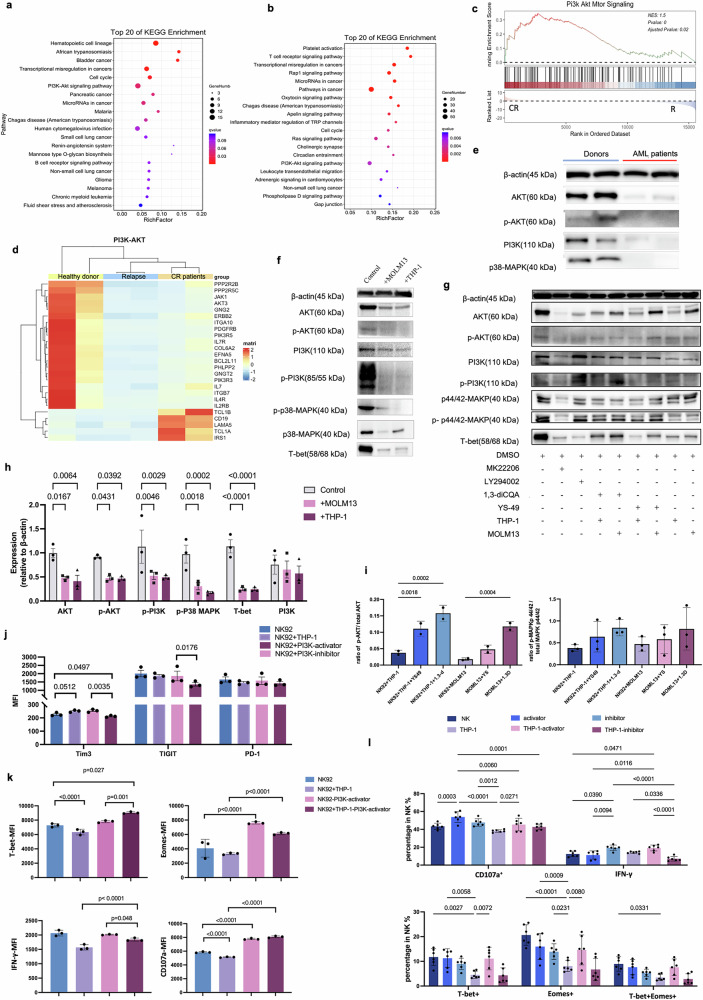
PI3K-AKT signaling pathway inhibition involved in NK exhaustion in AML patients (**a**, **b**) KEGG analysis of the DEGs between relapsed and CR patients (left) and healthy donors (right). **c** GSEA of PI3K-AKT-mTOR signaling in CR and relapsed patients. **d** heatmaps of genes related to the PI3K-AKT pathway among the three groups. **e** Representative western blots showing AKT, PI3K, p-AKT, p-PI3K, p-MAPK, and β-actin expression in primary NK cells from relapsed AML patient, healthy donors. **f** Densitometric quantification of the ratios of the levels of proteins of interest (i.e., AKT, p-AKT, PI3K, p-PI3K, T-bet, and p38-MAPK) to the level of the β-actin protein in NK92 cells cocultured with MOLM13 or THP-1 cells. **g** Densitometric quantification of the ratios of NK cells treated with the PI3K-AKT inhibitor LY294002 or MK2206, or treated with the activator 1,3-diCQA or YS49 and cocultured with THP-1 or MOLM13 cells. **h** Relative expression of target proteins level of β-actin protein, representative results from 3 independent experiments are shown. **i** Relative expression of p-AKT or p-p44/42 of the total AKT and MAPK respectively when NK92 cell cocultured with THP-1 or MOLM13 cells stimulated with or without PI3K-AKT activators. **j** Expression of exhaustion markers on NK92, including Tim3, TIGIT and PD-1, treated with THP-1 cells, PI3K-AKT activators and inhibitors, NK92 alone were used as control. **k** Expression of T-bet, Eomes, IFN-γ and CD107a in exhausted NK92 cells supplemented with PI3K-AKT activators when cocultured with THP-1 cells. **l** Expression of exhaustion markers on primary NK cells, including Tim3, TIGIT and PD-1, in cells treated with THP-1 cells, PI3K-AKT activators and inhibitors. The results represent a composite of 3 independent experiments

To characterize the transcriptional profile of NK cells during AML relapse, we conducted RNA-seq analysis in healthy donors, CR patients, and relapsed AML patients. Overall, the gene expression patterns of the relapsed, CR, and HD groups were significantly different. KEGG enrichment analysis indicated that PI3K-AKT signaling was inhibited in relapsed patients compared to CR patients (Fig. 5a) and healthy donors (Fig. 5b). Furthermore, GSEA analysis also showed that PI3K-AKT signaling was inhibited in relapsed patients (Fig. 5c).

Previous studies have highlighted the essential role of p110δ, a subset of PI3K protein, for NK cell maturation and function.^44^ To addressed this, we examined the expression of genes related to the PI3K-AKT pathway of GO terms and found that genes were significantly downregulated in relapsed patients (Fig. 5d).

It remains unclear whether PI3K-AKT inhibition plays a key regulatory role during AML-induced NK cell exhaustion. Subsequently, we isolated NK cells from both relapsed patients and healthy donors for western blotting analysis to confirm total and phosphorylated protein expressions related to the PI3K-AKT pathway. The results found significantly lower levels of AKT and PI3K as well as reduced levels of phosphorylated AKT (p-AKT), phosphorylated PI3K (p-PI3K), and phosphorylated MAPK (p-MAPK) in AML patient-derived NK cells compared to control NK cells (Fig. 5e).

To answer the question whether NK cell exhaustion was correlated with the inhibition of PI3K-AKT pathway, NK92 cell were cocultured with AML cell lines for 5 days to assess the activation of PI3K-AKT pathway. Results showed that exhausted NK92 cells downregulated the p-AKT and p-PI3K pathways after coculturing with THP-1 or MOLM13 cells for 5 days in vitro (Fig. 5f–h), furthermore treatment of NK cells with PI3K-AKT inhibitor (MK2206 and LY294002) significantly decreased the p-AKT, p-MAPK and T-bet expression, while PI3K activators (1,3-diCQA and YS-49) was benefit for NK92 cells to relieve the inhibition of pathway induced by NK cells exhaustion, which increased the level of p-AKT significantly (Fig. 5g, i). Meanwhile, supplementation with a PI3K-AKT activator proved beneficial for enhancing NK92 cell against THP-1 activity, leading to increased cytokine release (Fig. 5j) and transcription factor expression while decreasing exhaustion markers (Fig. 5k).

Primary NK cells were co-cultured with THP-1 cells for 5 days at an effector-to-target (E:T) ratio of 5:1 to induce exhaustion. Subsequently, PI3K-AKT pathway modulators (either activators or inhibitors) were administered at optimized time points to evaluate their capacity to rescue NK cell functional activity. Results showed that NK cells downregulated the IFN-γ and CD107a and transcription factors Eomes and T-bet expression after the long-term stimulation by THP-1 cells, while timely addition of a PI3K activator can effectively improve primary exhausted NK cell function (Fig. 5l). In summary, the exhausted NK cells were associated with inhibition of the PI3K-AKT signaling pathway; meanwhile, PI3K-AKT activators could relieve the NK cell exhaustion.

### Interaction of NKG2A/HLA-E drives the PI3K/AKT inhibition in exhausted NK cells

**Fig. 6:**
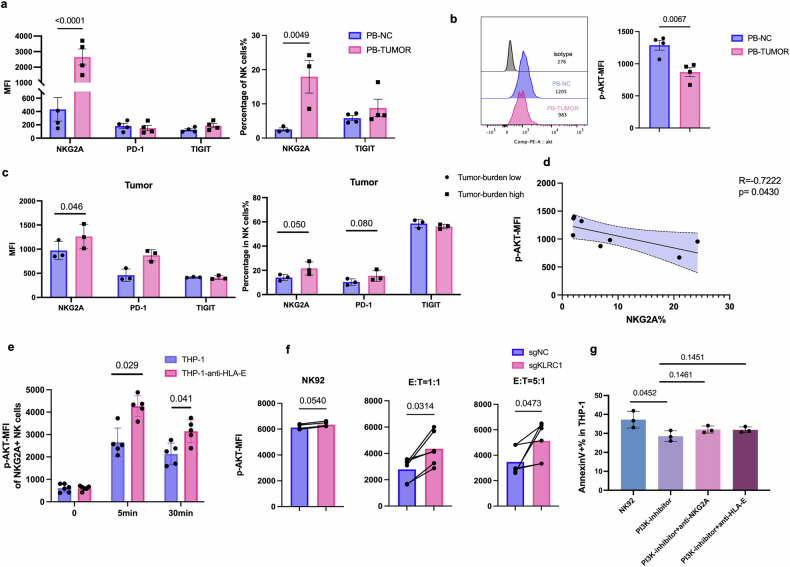
Interaction of NKG2A/HLA-E drives the PI3K/AKT inhibition in exhausted NK cells (**a**, **b**) The expression of NKG2A (**a**) and p-AKT (**b**) on PB NK cells between HL-60 tumor bearing mice and negative control at day 15. **c** The Frequency and MFI of NKG2A on NK cells from different tumor burden mice. **d** The correlation of NKG2A and p-AKT expression on HL-60 tumor bearing mice PB NK cells. **e** Anti-HLA-E treated THP-1 target cells increased P-AKT of primary NK cells after 5 days coculture and then stimulated with PMA and ionomycin for different time point. **f** NKG2A KO NK92 cells increased the p-AKT expression when cocultured with THP-1 cells under different E:T ratio of 1:1 and 5:1. **g** Anti-NKG2A and anti-HLA-E were unable to rescue the dysfunction of NK cells induced by the PI3K-AKT inhibitor (*N* = 3)

The question of whether excessive activation of the NKG2A/HLA-E pathway serves as a trigger for PI3K-AKT inhibition remains unclear. In the HL-60 tumor bearing mice, we noticed that peripheral blood NK cells from AML tumor bearing mice expressed higher NKG2A (Fig. 6a) and lower p-AKT expression (Fig. 6b) after 2 weeks AML stimulation. Moreover, we found that higher tumor burden was related to the overexpressed NKG2A (Fig. 6c). Furthermore, a significant negative correlation was observed between NKG2A expression levels and phosphorylated AKT levels (Fig. 6d), suggesting that the NKG2A/HLA-E axis may contribute to PI3K/AKT pathway suppression in exhausted NK cells.

It needed to determine that whether NKG2A/HLA-E axis blockade could restore the PI3K/AKT signaling pathway inhibition in exhausted NK cells. THP-1 cells were pretreated with an anti-HLA-E antibody and cocultured with NK92 cells for 5 days and then the p-AKT of NK cells were tested. Results shown than when the target cell’s HLA-E was blocked, the phosphorylation of AKT of NKG2A^+^ NK cells was significantly increased than untreated groups (Fig. 6e), suggesting that interfering with the NKG2A/HLA-E axis could prevent the inhibition of signaling pathways during NK cell exhaustion. Furthermore, we wondered that whether NKG2A KO could activate PI3K-AKT signaling pathway. Results showed that the knockout of NKG2A effectively enhanced the phosphorylation of the PI3K-AKT pathway (Fig. 6f). In order to answer whether PI3K-AKT is the downstream signaling pathway for the NKG2A/HLA-E axis, NK92 cells with a PI3K-AKT inhibitor overnight and then the anti-NKG2A or anti-HLA-E antibody were added to attempt to rescue the inhibition of NK cells. Our results found that neither anti-NKG2A nor anti-HLA-E was able to rescue PI3K-AKT inhibition on NK cells (Fig. 6g), indicating that PI3K-AKT was the downstream of NKG2A/HLA-E axis.

Collectively, our study determined that interfering the NKG2A/HLA-E axis can effectively reinvigorate NK cell exhaustion under tumor conditions which might be related to the recovery of PI3K-AKT signaling activation.

## Discussion

In this study, we have demonstrated that long-term AML cell stimulation induces NK cell exhaustion through the over interaction of HLA-E and NKG2A, resulting in the inhibition of the PI3K/AKT pathway. Previous studies have extensively addressed the dysfunction of NK cells with impaired antileukemic function in AML patients.^16–19^ The tumor microenvironment, which includes T regulatory cells, myeloid-derived suppressor cells (MDSCs), tumor-associated fibroblasts (TAFs) and TGF-β, significantly suppresses NK cell function and induces NK cell dysfunction in many tumors.^45^ Our findings revealed that NKG2A varies with the progression or remission of AML disease. NKG2A is an inhibitor receptor in NK and T cells, which has been reported overexpressed in various tumors.The NKG2A/HLA-E axis transmits classic inhibitory signals through receptor‒ligand interactions, interaction of NKG2A/CD94 axis by blocking the NKG2A enhances NK-mediated tumor cell killing in vitro.^34^ While it is uncertain that whether NKG2A/HLA-E will induce NK cells exhaustion in AML. Our study revealed that NK cells with high expression of NKG2A in relapsed AML patients post HSCT exhibited an exhaustion phenotype, along with weakened killing function and reduced expression of transcription factors. AML cells could directly induce the exhaustion of NK cells via the NKG2A/HLA-E axis, blocking NKG2A or HLA-E were both effectively protected NK from exhaustion, and increased the function of NK cells. Recently studies showed that knockout of NKG2A could further enhance the efficacy of primary NK cells^46^ or CAR-NK cells in clearing AML cells.^32^ In our study, we also found that NKG2A knockout was effective to elevate NK cells function to kill AML cell lines in vitro which decreased the LAG3 expression significantly. These findings suggest that therapeutic intervention targeting NKG2A, either through blockade or genetic knockout, may potentially alleviate NK cell exhaustion and restore NK cell functionality.

Tan et al.^26^ found that the PI3K-AKT pathway inhibition in NK cells leads to significant functional impairment, characterized by decreased cytotoxic activity and impaired cytokine production. Consistent with this finding, our study also observed the inhibition of the PI3K-AKT pathway in exhausted NK cells. Furthermore, we demonstrated that activators of the PI3K-AKT pathway could rescue NK cells exhaustion even under constant stimulation by leukemia cells. NKG2A knockout in NK92 cells significantly enhanced p-AKT levels, even following prolonged co-culture with THP-1 cells across various E:T ratios. Furthermore, we evaluated the therapeutic potential of YS-49, a PI3K-AKT pathway activator, in a CDX mouse model. However, the results were suboptimal, as YS-49-pretreated NK cells demonstrated limited anti-tumor efficacy, and oral administration of YS-49 posed potential risks of tumor cell activation,^47^ resulting in a shortened survival time of mice (supplementary Fig. 6a–c). We hypothesize that this phenomenon may be attributed to the dual effects of the activator on both tumor cells and NK cells. In our study, YS-49 functions as a PI3K/AKT activator (a downstream effector of RhoA), though this compound lacks isoform-selective specificity for PI3K. The class I PI3K family, a well-characterized group of lipid kinases, exhibits distinct expression patterns: PI3Kα and PI3Kβ are ubiquitously expressed in nucleated cells, whereas PI3Kγ and PI3Kδ display leukocyte-restricted expression. Notably, PI3Kγ and PI3Kδ isoforms are essential regulators of NK cell differentiation and effector functions,^44,48^ while class III PI3K governs NK cell maturation and senescence.^49^ Intriguingly, selective activation of PI3Kα has been shown to drive myocardial and neuronal regeneration,^50^ highlighting the therapeutic promise of isoform-directed PI3K modulation. Building on these insights, we propose that systematic CRISPR-Cas9 library screening could serve as a powerful strategy to pinpoint both classical and non-classical PI3K isoforms capable of reactivating exhausted NK cells, thereby amplifying their cytotoxic potential—a critical avenue for next-generation immunotherapies.

The overexpression of HLA-E can impede NK cell function by binding to the receptor NKG2A, leading adverse effects on NKG2A^+^ CD8^+^ T cells.^42^ Our study revealed elevated HLA-E expression on blast cells from relapsed AML patients. We hypothesized that this upregulation might be associated with inflammatory microenvironmental factors. Through in vitro experiments, we demonstrated that inflammatory cytokine cocktails significantly increased HLA-E levels on AML blast cells, potentially explaining the overactivated NKG2A/HLA-E axis observed in post-HSCT relapsed patients. This finding aligns with previous reports indicating that inflammatory tumor microenvironments contribute to immune cell exhaustion. Notably, serum IL-10 was shown to upregulate both TIGIT and NKG2A expression on NK cells.^24^ The activation of the IFNγ signaling pathway is associated with venetoclax resistance in AML, in which the majority of IFNγ secretion comes from T cells and NK cells in AML patients.^43^ A limitation of this study is the absence of direct experimental evidence linking NK cell exhaustion to inflammatory cytokine exposure in relapsed AML. Future studies should systematically investigate whether prolonged cytokine signaling directly drives exhaustion-related phenotypic and functional alterations in NK cells. The anti-NKG2A has been reported failed to control tumor progression while the neoadjuvant with durvalumab is effective to in lung cancer.^51^ In addition, the combination of anti-NKG2A and PD-L1 was efficient to overcome the PD-L1 resistant triple negative breast cancer (TNBC) with the synergistic effect to elimination both MHC-I positive and MHC-I negative tumor cells.^52^ In our CDX mouse model, the combination of anti-NKG2A and anti-PD-1 antibodies demonstrated initial efficacy in delaying tumor progression during early stages, but failed to maintain tumor control in later phases (supplementary Fig. 7). We hypothesize that this limited efficacy may result from poor NK cell persistence in vivo and low PD-1 expression levels on NK cells. Importantly, our study identified NKG2A knockout in AML-derived NK cells as a promising strategy to overcome NK cell exhaustion. These genetically modified NK cells exhibited superior anti-tumor efficacy than controls both in vitro and in vivo. It is reported that the deletion of NKG2A enhanced the activation of NK cells and the antibody-dependent cell-mediated cytotoxicity(ADCC) against tumor cells.^46^ Meanwhile, the suppressive signaling pathways were downregulated in NKG2A deleted NK cells. In summary, these results indicated that NKG2A deficiency or NKG2A blockade could enhance anti-leukemia immunity by reversing the exhaustion of NK cells, at least partially by restoring the activation of PI3K-AKT pathway. Our findings underscored the need for further investigation to determine whether NKG2A inhibition or gene editing alone or in combination with other immunotherapeutic approaches to optimize therapeutic strategies for AML.

## Materials and methods

### Human sample collection and cell lines

Bone marrow mononuclear cells (BMMCs) were collected from 12 healthy donors (HD group), 30 relapsed AML patients post-HSCT (relapse group), and 30 AML patients who achieved complete remission post-HSCT (CR group) from May 2018 to November 2020 at the Peking University People’s Hospital. The diagnosis of AML, CR or relapse, as well as donor selection and stem cell harvesting, was performed in accordance with previous reports.^53,54^ The clinical characteristics of the patients who experienced relapse or remission were compared in Supplementary Table 1. NK92 cells were generously provided by Professor Zhigang Tian at the Chinese Academy of Sciences, and K562, THP-1, YT, Kasumi-1, OCI-AML2 and MOLM13 cells were obtained from the ATCC. The Cas9 protein: sgRNA ratio for the THP-1 cell to knockout HLA-E was 1:1.2, the voltage for AML cell lines nucleofection was 480 V. AML cell lines with or without HLA-E were utilized for in vitro tests. All cells underwent rigorous identification and mycoplasma testing to ensure there was no chlamydia infection present in our study. This study was approved by the ethics committee of the Peking University People’s Hospital and the written informed consent was obtained from all patients prior to study entry in accordance with the Declaration of Helsinki. Ethics approval number and consent to participate and the approval number is 2017PHB013-01.

### Antibodies and flow cytometric analysis

The profiles of NK cells (including CD57, KIRs, NKp30, NKp46, NKG2D, NKG2C, NKG2A, PD-1, TIM-3, TIGIT, EOMES, and T-bet), NK cell ligands expressed on blast or normal CD34 + cells (including HLA-A, HLA-B, and HLA-C, HLA-E, MICA/B, PD-L1, ULBP 2/5/6, CD155, CD112), and the phosphorylation of AKT in NK cells were analyzed by flow cytometry according to the manufacturer’s instructions. CD34+ cells were gated as SSA^-^CD45^dim^ CD34^+^ cells as previously described.^55^ Gating strategy of CD34+ cells and the expression level of ligands were shown in Supplementary Fig. 4. Isotype-matched controls were used. In the intracellular cytokine detection experiment, protein transport inhibitors and CD107a antibodies were added prior to incubation. Cells were collected 6 h later and manipulated in accordance with the instructions of intracellular cytokine detection reagents. Before cell collection, live/dead dye was added, incubated at 37 °C for 30 min. Cells were pretreated with Fc-blocking reagent, fixed and permeabilized with phospho-flow buffer (BD Biosciences) and washed with staining buffer (BD Biosciences) as suggested by the manufacturer. Cell gating analysis was performed by flow cytometry (LSRFortessa, BD Biosciences) and FlowJo software. The antibodies used for flow cytometry are listed in Supplementary Table 2.

### Cytotoxicity assay

Peripheral blood mononuclear cells (PBMCs) or BMMCs (1 × 10^6^ cells/mL) from healthy donors, AML CR patients and relapsed patients, were isolated with Ficoll and NK cells were by negative selection with MACS according to the manufacturer’s instructions. AML blast cells were defined as SSA^-^CD45^dim^ CD34^+^ cells, and sorted by flow cytometry from the primary AML patients as target cells in the killing assay. The apoptosis of AML blast cell was evaluated after cocultured with NK cells from different groups for 4, 24 and 48 h at an E:T ratio of 1:1, 5:1, 10:1 and AML blast cells alone in NK cell medium were used as control (Supplementary Fig. 1c). 1000 U/mL IL-2 was added every two days in each culture system, CD107a, TNF-α, IFN-γ, granzyme B, and perforin were assessed by multiparametric flow cytometry. Target cells cocultured with NK92 or primary NK cells to induce NK cells exhaustion, after that NK cells were sorted by flow cytometry for cytotoxicity function assay.

### RNA sequencing (RNA-seq)

For bulk RNA sequencing analysis, NK cells were isolated from relapsed AML patients, CR, and healthy donors by flowcytometry cell sorting system. Differentially expressed gene analysis was performed with the R package DESeq2. Pathway enrichment of DEGs was analyzed by Kyoto Encyclopedia of Genes and Genomes (KEGG) and gene set enrichment analysis (GSEA). The raw and processed data generated in this study are publicly available in Gene Expression Omnibus (GEO) at GSE252702 (https://www.ncbi.nlm.nih.gov/geo/query/acc.cgi?acc=GSE252702).

### Cell isolation and generation of *KLRC1* KO NK cells

NK92 cells were cultured in NK92MI medium supplemented with 200 IU interleukin (IL)-2. Primary NK cells isolated from PBMC of healthy donor or from BMMC of AML patients by negative selection with MACS according to the manufacturer’s instructions. The NKG2A^+^NKG2C^-^ NK subpopulation for gene editing to knockout the *KLRC1* was sorted by flow cytometry, in which NK cells were expanded ex vivo by membrane-bound IL-21/4-1BBL-expressing K562 cells as previously described.^56^ NKG2A knockout NK92 or primary NK cells were constructed via nucleofection by RNP system. Cas9 recombinant protein was purchased from Thermo Fisher (5 µg/ml), and the sgRNAs for *KLRC1* are listed in Supplementary Table 2. The nucleofection assay was performed by Celetrix LE^+^(Celetrix LLC, USA).The Cas9 protein: sgRNA ratio for the NK92 cell or primary NK cells was 1:1.2, the voltage for NK92 cell nucleofection was 460 V, and that for primary NK cells was 540 V. The purity of NKG2A^KO^ NK cells were achieved nearly 100% after sorted by flow cytometry for further assessment. NK cells with or without NKG2A expression were utilized for in vivo or in vitro tests. All primary NK cells were cultured in GT-T551 H3 medium with 1000U/ml IL-2 and maintained at 37 °C under 5% CO_2_ atmosphere for the experiments.

### Treatment of NK cells in vitro and western blot analysis

NK cell lines (YT or NK92) were cocultured with AML cell lines (K562, THP-1, Kasumi-1, OCI-AML2 and MOLM13) at an E:T ratio of 10:1 for 5 days under the NK92 culture medium for NK92 cells (with 200U/ml IL-2) or RPMI1640 contained with 10% FBS for YT cells. Fresh medium supplemented with 200 U/ml IL-2 was added every other day. Primary NK cells from relapsed, CR patients and healthy donors were isolated as previous described. NK cells were cocultured with THP-1 or AML blasts cells at different E:T ratios for 10 days with the GT-T551 H3 culture medium plus 1000 U/mL IL-2. Fresh culture medium with 200 U/ml IL-2 were added every other day. The NK cell phenotypes, functional characteristics, and associated transcription factors were evaluated finally, and western blot analysis was performed. AML blasts were cultured with IFN-γ (500 ng/mL; PeproTech), rIL-10 (10 ng/mL; PeproTech) or TNF-α (10 ng/mL; PeproTech). NK cells were incubated with an anti-NKG2A antibody (0.25 µg/1 × 10^6^ cells, R&D), and THP-1 cells were incubated with an anti-HLA-E antibody (10 µg/ml, Thermo Scientific) for 1 h before coculture. The PI3K-AKT activator 1,3-dicaffeoylquinic acid (10 μM) or YS-49 (10 μM) and the PI3K-AKT inhibitor MK2206 (10 μM) was added during coculture for 8–12 h, DMSO was used as a control. NK92 cells were sorted for western blot analysis after coculture for 5 days. Meanwhile, exhausted NK92 cells were treated with the PI3K-AKT activator YS-49 for 8–12 h, and cytotoxicity assays against K562 were performed at E:T ratio of 5:1.

For the NKG2A knockout NK cells, NKG2A KO NK92 cells or primary NKG2A KO NK cells were then cocultured with Thp-1 cells for 5 days to induce NK cell exhaustion and NKG2A^+^ with nonsense sgRNA nucleofection were used as control. NK92 or primary cells cytotoxicity were assessed by stimulated with K562 target cells under the E:T ratio =5:1, cytokine release and phosphorylation of AKT of NK cells were assessed by flow cytometry.

### In vivo experiments with mouse leukemia models

Female NOD.Cg-Prkdcscid Il2rgtm1^Vst/Vst^ (NPG) mice, aged 7 weeks and weighing 19–21 g, were obtained from Beijing Vital Star Biotechnology Company. Luciferase-expressing THP-1 cells (1× 10^5^) or vehicle alone (PBS) were injected intravenously (i.v.), and 7 days later, PBS or expanded NK cells (1× 10^7^) were injected once per week for 2 total injections. NK cells were expanded ex vivo by membrane-bound IL-21/4-1BBL-expressing K562 cells as previously described. An anti-NKG2A blocking antibody (200 µg/mouse, Monalizumab, MCE) was infused with NK cells in 3 doses (i.v.). In another in vivo experiment, AML patient-derived expanded NK cells with or without NKG2A knockout were infused into the THP-1-luciferace mice models. 50000 U of human recombinant IL-2 per mouse was injected intraperitoneally (i.p.) every two days to maintain infused NK cells survival. Peripheral blood was collected every two days to detect the in vivo persistent and exhaustion markers of NK cells in each group. In the HL-60 subcutaneous xenograft mouse model, 1× 10⁵ HL-60 cells were inoculated into the dorsal thoracic region of mice. On day7 and day15 post-engraftment, mice were sacrificed to collect NK cells from the spleen, peripheral blood, and tumor tissues for phenotypic analysis. Age-matched mice without tumor inoculation served as controls. All mice were maintained in an SPF-level environment, which is in compliance with animal ethics. All Animal experiments were approved by an ethical committee No.BCAA0305.

### Statistical analysis

Analyses were performed with SPSS statistics version 26.0. We used the chi-squared test or Fisher’s exact test for categorical variables, the Mann–Whitney U test for continuous variables, and one-way ANOVA for multigroup comparisons. The data were shown as the mean ± standard deviation. The survival time among groups were analyzed by log-rank test. *P* < 0.05 was considered statistically significant. The schematic diagram generated from online tools bioRender (https://www.biorender.com).

## Supplementary information

Supplementarymaterials

## Data Availability

All raw and processed data sets generated and used in this study are available upon request from the corresponding author, Xiang-Yu Zhao (zhao_xy@bjmu.edu.cn).
