# Supplementary material for: Overexpressing natural killer group 2 member A drives natural killer cell exhaustion in relapsed acute myeloid leukemia
Source: Signal Transduct Target Ther. 2025 May 5;10:143. doi: 10.1038/s41392-025-02228-5 (PMC12050333; doi:10.1038/s41392-025-02228-5)
Supplement: Supplementary file 1 — Supplementarymaterials [file 41392_2025_2228_MOESM1_ESM.docx]

Supplementary Materials for

**Overexpressing Natural Killer Group 2 Member A drives natural killer cell exhaustion in relapsed acute myeloid leukemia**

Juan Xie ^1#^, Xue-Fei Liu^1,3#^, Tong Zhou^1#^, Long Liu^1^, Rui-Qin Hou^2^, Xing-Xing Yu^1,3^, Ze-Ying Fan^1^, Qian-Nan Shang^1^, Ying-Jun Chang^1^, Xiao-Su Zhao^1^, Yu Wang^1^, Lan-Ping Xu^1^, Xiao-Hui Zhang^1^, Xiao-Jun Huang^1,3*^, Xiang-Yu Zhao^1,3*^

Correspondence to:Xiang-Yu Zhao (zhao_xy@bjmu.edu.cn )

**This PDF file includes:**

Materials and Methods

Figures. S1 to S7

Tables S1 to S2

**Supplementary Materials and Methods ：**

Female NOD. Cg-Prkdcscid Il2rgtm1^Vst/Vst^ (NPG) mice (7 weeks, 19-21 g) were purchased from Beijing Vital Star Biotechnology Company. Luciferase-expressing THP-1 cells (1× 10^5^) or vehicle alone (PBS) were injected intravenously (i.v.), and 7 days later, PBS or expanded NK cells (1 × 10^7^) were injected once per week for 2 total injections. NK cells were expanded ex vivo by membrane-bound IL-21/4-1BBL-expressing K562 cells as previously described. The activator YS-49 (50mg/kg) and MK2206(60mg/kg) were given by intragastric administration when NK cells infused. In addition, the YS-49(10uM/10^6^ cells/ml) and MK2206(10uM/10^6^ cells/ml) were pretreated with NK cells for 4hours before NK cells infusion(Figure. S6). An anti-NKG2A blocking antibody (200 µg/mouse, Monalizumab, MCE) and anti-PD-1antibody(200 µg/mouse, Pembrolizumab, MCE) was co-infused with NK cells in 3 doses (i.v.) (Figure. S7).

 Figure. S1.

Figure. S1. (a) gating strategy of NK phenotypes in healthy donors, CR and relapsed patients. (b) The frequency and MFIs of activation receptors expressed on BMNK cells from healthy donors(HD),complete remission patients(CR) and relapsed patients(Relapse)post HSCT. (c) the representative figure of the apoptosis in AML blast cells induced by NK cells.

Figure. S2.

Figure. S2. (a) T-bet+eomes+ NK92 cells co-cultured with or without THP-1 cells for 5days under E:T=5:1.(b) Representative frequency of NKG2A expression with or without THP-1 cells for 5 days under E:T=5:1.

Figure. S3

Figure. S3. (a) gating strategy of CD34+ cell for ligand analysis (b) Representative frequency of the MFIs of NKG2D ligands, Including ULBP-2/5/6, MIC A/B and the ligands of TIGIT expressed, including CD155 and CD112 expressed on AML blast cells from healthy donors (HD), complete remission patients(CR) and Relapsed patients(Relapse) .

Figure. S4.

Figure. S4. NK cell receptor ligands (NKRLs) and the inflammatory environment of plasma. (a)MFI of HLA-ABC expression in CD34+ cells from HDs (n=5), CR patients (n=10) and relapsed patients (n=8). (b and c) HLA-E(b) and PD-L1(c) expression in CD34+ cells from HDs, CR patients or relapsed patients. (d) the MFIs of NKG2D ligands, Including ULBP-2/5/6, MIC A/B and the ligands of TIGIT expressed, including CD155 and CD112 expressed on AML blast cells from healthy donors(HD), complete remission patients(CR) and Relapsed patients(Relapse). (e)Differences in HLA-ABC expression in LSCs between patients with relapsed AML and those with primary AML (n=22). (f and g) Comparison of HLA-E (f) and PD-L1(g) expression in LSCs between relapsed patients and primary AML patients. (h) The concentrations of cytokines (i.e., IL-10, TNF-α, and IFN-γ) in BM plasma samples from HDs (n=5) and CR (n=20) and relapse (n=20) patients. (i) The frequency and MFI of HLA-E expression in AML blasts sorted from AML patients after stimulation with IL-10 (10 ng/ml), TNF-α (500 ng/ml), and/or IFN-γ (500 ng/ml) in vitro for 5 days. The results represent a composite of 4 independent experiments with blasts sorted from 4 AML patients. (j) cytokine concentration in plasma among healthy donors (HD), complete remission patients(CR) and Relapsed patients (Relapse).

Figure. S5.

Figure. S5. (a) the LAG3+ expression on primary NK cell between sg KLRC1 and NC group. (b) representative frequency of the apoptosis of THP-1 cells induced by NK cells with NKG2A KO or not. (c) the animal living imaging at different time points among THP-1, THP-1,-NK and THP-1-NKG2A ^KO^ NK group.(N=5)

Figure. S6.

Figure. S6. (a)the animal living imaging at different time points between Thp-1-luci+NK+activator and Thp-1-luci+NK+inhibitor groups, in which the PI3K-AKT inhibitors or activator were given by intragastric administration.(N=12)；(b) survival curve of among 4 groups. (c) the animal living imaging at different time points among Thp-1-luci,Thp-1-luci -NK,Thp-1-luci-activator, in which the PI3K-AKT inhibitors or activator were pretreated on NK cells in vitro before NK cell infusion.(N=12)

Figure. S7.

Figure. S7. (a)the animal living imaging at different time points in THP-1-NK-anti-NKG2A-anti-PD-1 combination group.(N=12)

Table S1. Patients' and healthy donor's characteristics

**Supplementary Table1. Patients' and healthy donor's characteristics**

| Patient Characteristics | Relapsed Patients | Complete Remission  Patients | P Value | Healthy Donors |
| --- | --- | --- | --- | --- |
| Number of patients | 30 | 30 |  | 12 |
| Gender |  |  | 0.292 |  |
| Male | 21/30 | 17/30 |  | 6 |
| Female | 9/30 | 13/30 |  | 6 |
| Age, years (median) | 39 (8-64) | 33 (17-51) | 0.487 | 41 (28-55) |
| Disease: AML | 30 | 30 | 0.705 |  |
| AML without maturation | 1/30 | 1/30 |  |  |
| AML with maturation | 4/30 | 5/30 |  |  |
| Acute myelomonocytic leukemia | 5/30 | 5/30 |  |  |
| Acute monoblastic/monocytic leukemia | 7/30 | 6/30 |  |  |
| AML with BCR::ABL1 fusion | 0/30 | 1/30 |  |  |
| AML with RUNX1::RUNX1T1 fusion | 2/30 | 3/30 |  |  |
| AML with CBFB::MYH11 fusion | 0/30 | 2/30 |  |  |
| AML with NPM1 mutations | 6/30 | 3/30 |  |  |
| AML with CEBPA mutations | 1/30 | 3/30 |  |  |
| AML-MR | 4/30 | 1/30 |  |  |
| Mutation profile |  |  |  |  |
| FLT3-ITD | 7/30 | 1/30 | 0.052 |  |
| TP53 | 1/30 | 0/30 | >0.999 |  |
| c-Kit | 1/30 | 1/30 | >0.999 |  |
| Complex karyotype | 10/30 | 2/30 | 0.021 |  |
| Remission status pretransplant |  |  | 0.8 |  |
| CR1 | 26/30 | 23/30 |  |  |
| ≥CR2 | 2/30 | 6/30 |  |  |
| NR | 2/30 | 1/30 |  |  |
| Donor type |  |  | 0.191 |  |
| Unrelated donor | 1/30 | 1/30 |  |  |
| Identical siblings | 9/30 | 6/30 |  |  |
| Haploidentical related donor | 20/30 | 24/30 |  |  |
| ABO |  |  | 0.8 |  |
| Match | 15/30 | 16/30 |  |  |
| Mismatch | 15/30 | 14/30 |  |  |
| Median MNCs of grafts, *10^6^/kg (range) | 8.62  (5.09-13.45) | 9.18  (4.68-17.27) | 0.316 |  |
| Median CD34* of grafts, *10^6^/kg (range) | 2.59  (1.00-5.20) | 2.83  (0.69-9.26) | 0.542 |  |
| Ratio of AML blast cells in BMNC (range) | 19.62  (5-76) | 0.00 | / |  |
| Acute GvHD at BM collection |  |  | / |  |
| None | 30/30 | 30/30 |  |  |
| Grade 0-IV | 0/30 | 0/30 |  |  |
| Chronic GvHD at BM collection |  |  | 0.723 |  |
| None | 26/30 | 25/30 |  |  |
| With cGvHD | 4/30 | 5/30 |  |  |
| CMV infection at BM collection |  |  | / |  |
| With CMV infection | 30/30 | 30/30 |  |  |
| Without CMV infection | 0/30 | 0/30 |  |  |
| Median post-transplant days at BM  collection (range) | 331 (54-885) | 399 (82-1034) | 0.487 |  |

AML, acute myeloid leukemia, GvHD graft-versus-host disease, HSCT hematopoietic stem cell transplant, CMV cytomegalovirus

*Clinically relevant CMV reactivation is defined as a CMV reactivation requiring systemic preemptive antiviral treatment accordance to institutional guidelines (DNAemia >1000 cp/ml, measured on plasma)

Table S2. antibody and sgRNA sequence list

| No | AntibodyTarget | Conjugate | Company | cat |
| --- | --- | --- | --- | --- |
| 1 | CD3 | APC-H7 | BD Pharmingen | 560176 |
| 2 | CD3 | BV510 | Biolegend | 300448 |
| 3 | CD3 | Percp | Biolegend | 344814 |
| 4 | CD56 | BUV737 | BD Pharmingen | 564447 |
| 5 | CD56 | PE | Biolegend | 304606 |
| 6 | CD56 | APC-CY7 | Biolegend | 318332 |
| 7 | CD57 | PERCP | Biolegend | 359622 |
| 8 | CD57 | BV605 | Biolegend | QA17A04 |
| 9 | CD57 | FITC | BD Pharmingen | 663497 |
| 10 | CD158a | FITC | BD Pharmingen | 339504 |
| 11 | CD158b | FITC | BD Pharmingen | 559784 |
| 12 | NKB1 | FITC | BD Pharmingen | 555966 |
| 13 | NKG2A | PECY7 | MASC | 130113567 |
| 14 | NKG2D | PE | Biolegend | 320806 |
| 15 | NKG2C | AF700 | R&D | FAB138N-100 |
| 16 | PD-1 | PE/Dazzle 594 | Biolegend | 329940 |
| 17 | PD-1 | BV421 | Biolegend | 367422 |
| 18 | TIM-3 | APC | Ebioscience | 17310942 |
| 19 | TIGIT | BV605 | Biolegend | 372712 |
| 20 | IFN-γ | BV510 | Ebioscience | 502544 |
| 21 | IFN-γ | PE | Biolegend | 506507 |
| 22 | IFN-γ | BV421 | Biolegend | 502532 |
| 23 | CD107a | BV786 | BD Pharmingen | 563896 |
| 24 | CD107a | BV421 | Biolegend | 328626 |
| 25 | CD45 | V500 | BD Pharmingen | 662912 |
| 26 | CD45 | APC-CY7 | Biolegend | 368516 |
| 27 | CD45 | BV650 | BD Pharmingen | 563717 |
| 28 | NKP46 | BV786 | BD Pharmingen | 563329 |
| 29 | NKP30 | BV421 | BD Pharmingen | 563385 |
| 30 | MCD45 | Percp | Biolegend | 103130 |
| 31 | TNF-α | AF700 | Biolegend | 502928 |
| 32 | GranzymeB | BV510 | BD Pharmingen | 563388 |
| 33 | Perforin | APC-CY7 | BD Pharmingen | 556437 |
| 34 | Perforin | PE | BD Pharmingen | 556437 |
| 35 | Ki-67 | PE | BD Pharmingen | 556027 |
| 36 | DAPI | Ultraviolet laser | Biolegend | 422801 |
| 37 | CD34 | BV421 | Biolegend | 343610 |
| 38 | CD13 | BV605 | Biolegend | 301728 |
| 39 | PD-L1 | BV650 | BD Pharmingen | 563740 |
| 40 | PD-L1 | PE | Biolegend | 329706 |
| 41 | CD117 | BV786 | BD Pharmingen | 743608 |
| 42 | MICA/B | FITC | Biolegend | 320912 |
| 43 | CD112 | PerCP | Biolegend | 337416 |
| 44 | HLA-E | PE | MACS | 130117550 |
| 45 | CD33 | PE-CF594 | BD Pharmingen | 562492 |
| 46 | CD155 | PE-Cy7 | Biolegend | 337614 |
| 47 | ULBP2/5/6 | APC | R&D | FAB12398A |
| 48 | CD38 | AF700 | Biolegend | 397106 |
| 49 | HLA-ABC | APC-Cy7 | Biolegend | 311426 |
| 50 | Annexin V | PE | BD Pharmingen | 51-65875X |
| 51 | 7-AAD | Percp | BD Pharmingen | 51-68981E |
| 52 | mCD49b | buv395 | BD Pharmingen | 740250 |
| 53 | mNKG2A | APC | Biolegend | 142807 |
| 54 | mTIGIT | PE/Dazzle™ 594 | Biolegend | 142109 |
| 55 | mPD-1 | BV421 | Biolegend | 135217 |
| sg RNA seqence | | | | |
| 1 | sg KLRC1-1 | GAAGCTCATTGTTGGGATCC | | |
| 2 | sg KLRC1-2 | AACAACTATCGTTACCACAG | | |
| 3 | sg KLRC1-3 | TGAACAGGAAATAACCTATG | | |
| 4 | sg HLA-E-1 | GTTGTCGAAGCGCACGAACT | | |
| 5 | sg HLA-E-2 | GAAAATCTGTGCGGTGTCCC | | |
| 6 | sg HLA-E-3 | CTCCGAGAGGAGTAAAAGGA | | |
